# An unusual appearance of a serous ovarian cyst coexisting with endometriosis: A case report

**DOI:** 10.1016/j.ijscr.2020.01.045

**Published:** 2020-02-06

**Authors:** Antoine Naem, Lamia Kouba, Bashar Al-Kurdy

**Affiliations:** a Faculty of Medicine of Damascus University, Damascus, Syria; b University Hospital of Obstetrics and Gynecology in Damascus, Syria

**Keywords:** IL, interleukin, TNF-α, tumor necrosis factorα, TGFβ, transforming growth factorβ, LH, luteinizing hormone, Case report, Endometriosis, Fibrosis, Serous cyst, Ovarian cyst, Adhesions

## Abstract

**Introduction:**

Endometriosis is an estrogen-dependent disease that can provoke fibrosis through the elevated concentration of TGF-β. Endometriosis-related fibrosis often manifests as pelvic adhesions, and it is almost always observed microscopically when examining the endometriotic lesions. Here we are reporting, for the first time in literature, an unusual gross appearance of a serous ovarian cyst that coexisted with endometriosis.

**Presentation of case:**

A 32-year-old G1P2 woman presented to our hospital complaining of the inability to conceive. She was scheduled to have an exploratory laparoscopy. Upon reaching the abdominal cavity, several islands of endometriosis and a left ovarian mass were spotted. The ovarian mass was resected by an excisional biopsy and the pathologic examination showed a benign ovarian cyst with extreme fibrosis in its wall.

**Discussion:**

Endometriosis often induces a chronic pelvic inflammation, mediated mainly by the over-activated macrophages. These cells secrete many cytokines and growth factors that alter the normal pelvic milieu. TGF-β levels are often elevated within the peritoneal fluid, which stimulate fibroblasts and provoke tissue fibrosis. One study suggested that ovarian stromal fibrosis can induce infertility by impairing folliculogenesis. LH-inhibiting regimens can reverse the stromal fibrosis, and improve the fertility outcomes.

**Conclusion:**

The mural fibrosis seen in the cystic wall reflects unrecognized ovarian stromal fibrosis induced by the fibrogenic effects of endometriosis. This process may play a potential role in provoking infertility in patients with endometriosis, and could be reversed by administrating the LH-inhibiting regimens.

## Highlights

• Fibrosis is a significant complication of endometriosis, provoked mainly by TGF-β.
• Endometriosis-induced fibrosis may alter the known morphology of the ovarian cysts.
• The mural fibrosis may reflect unrecognized fibrosis within the ovarian stroma.
• Endometriosis may have potential fibrotic effects on the ovarian stroma.
• Ovarian stromal fibrosis could contribute to the endometriosis-related infertility.

## 1 Introduction

Endometriosis is an estrogen-dependent disease defined by the presence of endometrial tissue, including endometrial glands and stroma, outside the uterine cavity. The pathogenesis of these ectopic islands isn’t fully understood yet, but the widely accepted hypothesis is the retrograde menstruation phenomenon, where the menstrual blood – that contains viable endometrial fragments – travels backwards through the fallopian tubes into the peritoneal cavity, where it implants and invades the peritoneum [1]. These lesions affect 25%–50% of infertile women [2]. In addition, a positive family history for endometriosis has been identified as a significant risk factor, because these women were found to be at 7 times higher risk of developing endometriosis in their lives [3]. Patients suffering from endometriosis often complain of a wide range of symptoms, and the most commonly reported manifestations are dysmenorrhea, dyspareunia, dyschezia, and infertility. Endometriosis can cause infertility either by enhancing aberrant genetic expression; that reduces the endometrial receptivity and inhibits progesterone [2], or through the chronic sterile inflammation mediated by the over-activated macrophages. These cells assist endometriosis at different pathogenesis phases, and secrete multiple inflammatory mediators that negatively affect the pelvic organs [4]. Furthermore, the chronic inflammation may cause chronic salpingitis [5], or generate peritoneal adhesions – mainly through the activity of TGF-β [6] – and ultimately disturb the normal reproductive function. In our case, the endometriosis-induced fibrosis manifested as extreme fibrous deposits within the stroma of a serous ovarian cyst. These fibrotic changes gave the cyst the shape of rough solid mass, and remarkably reduced its elasticity. These findings confused the cyst with other ovarian tumors such as fibromas and thecomas. To the best of our knowledge, this is the first case in literature that describes such unusual appearance of a serous ovarian cyst and discusses the potential pathways that led to its rare morphology and their possible effects on the folliculogenesis and female fertility. This work has been reported in line with the SCARE criteria [7].

## 2 Presentation of case

**Fig. 1:**
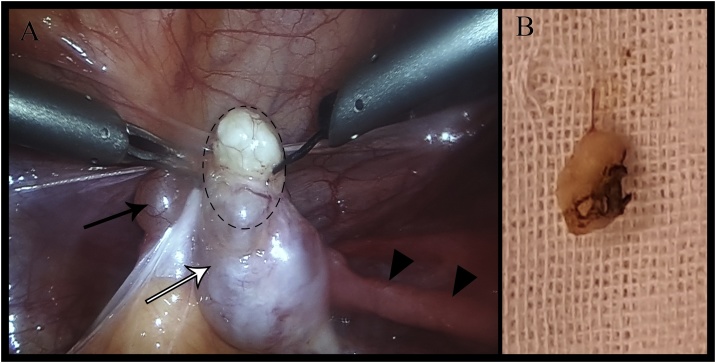
A) View from the surgical monitor showing an ovarian mass (Discontinuous circle), the left ovary (white arrow), a paratubal cyst (black arrow) and the left fallopian tube (small arrows). B) The gross appearance of the excised biopsy.

**Fig. 2:**
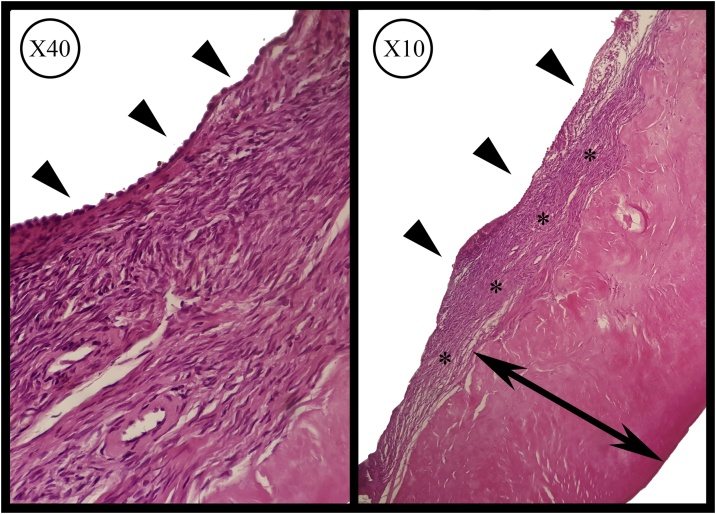
The microscopic appearance of the excised biopsy stained in Hematoxylin & Eosin. Image on the right shows the intensive fibrosis within the cyst’s wall (Double-headed arrow) and the relatively hypercellular cystic stroma (*). Image on the left shows the epithelial lining of the cyst (Arrow heads).

A 32-year-old G1P2 woman presented to the fertility clinic of our hospital complaining of the inability to conceive. The patient was in a good general status, and her medical history was unremarkable. Her menstrual cycle and her husband’s semen analysis were normal. Her serum gonadotropin, thyroid stimulating hormone, and Anti-Müllerian hormone levels were within normal limits. The uterus and both adnexa uteri were unremarkable upon transvaginal ultrasonography examination. The patient was scheduled for an exploratory laparoscopy to assess the pelvis and the reproductive organs. Upon reaching the abdominal cavity, several islands of endometriosis were spotted near the infundibulopelvic and uterosacral ligaments. A left ovarian mass and a small paratubal cyst in the left mesosalpinx were also found (Fig. 1.A). The ovarian mass was round, white, and firm upon palpation. Many adhesions were also observed. The direct inspection of the uterus and fallopian tubes coupled with the injection of the methylene blue solution transvaginally, revealed a bilateral obstruction of the fallopian tubes. An excisional biopsy of the ovarian mass was performed and sent for pathologic examination. The endometriotic islands were ablated and adhesiolysis was performed using monopolar electrocoagulation. The right fallopian tube was dilated successfully, unlike the left one, so a left salpingostomy was performed. The laparoscopy was thus completed, and the patient’s recovery was uneventful. The gross inspection of the ovarian mass revealed a cavitated rough mass measuring 1 cm in length and 0.5 cm in width (Fig. 1.B). The microscopic examination showed a benign serous ovarian cyst with intensive fibrosis within the stroma of its wall (Fig. 2). The patient will be followed-up by serial ultrasound to detect any future recurrence.

## 3 Discussion

Endometriosis is an estrogen-dependent lesion characterized by the presence of endometrial glands and stroma outside the uterine cavity. Its exact pathogenesis isn’t fully understood yet, but the most accepted hypothesis is the retrograde menstruation phenomenon, where the viable endometrial cells travel backwards with the menstrual blood through the fallopian tubes into the peritoneal cavity [1]. These cells are able to survive within the abdominal cavity due to the impairment of the patient’s immune response, as it fails in recognizing the abnormal localization of the endometrial cells and subsequently, fails to remove them [2]. Additionally, many cellular components of the immune system – especially macrophages – are accused of assisting the pathogenesis of endometriosis at different phases, such as promoting angiogenesis and cellular attachment to the peritoneum [4]. However, the most important contributors to the previously described events are the over-activated macrophages, which form the major cellular component of the peritoneal fluid in patients suffering from endometriosis, and secrete many inflammatory cytokines and growth factors [8], such as IL-1, IL-8, TNF-α, and TGF-β. However, more recent studies have introduced tissue fibrosis as another important feature of endometriosis, because it's observed microscopically nearly in all endometriotic lesions – even when the endometrial glands and stroma are absent – [9]. It’s suggested that the leading factor in the previously described phenomenon is the TGF-β as it is a chemotactic agent that recruits fibroblasts and activates them, while inhibiting the fibrinolysis mechanisms by upregulating the expression of tissue inhibitors of matrix metalloproteinase and downregulating the expression of matrix metalloproteinase [6]. These effects can lead to extensive fibrous deposits within the extracellular matrix. Indeed, fibrosis is often observed in-situ during the microscopic examination of the peritoneal lesions, or manifests macroscopically in pelvic adhesions [9], but in our case, it was present in the wall of an ovarian cyst far from the endometriotic islands. The cyst’s morphology was extensively modified by the endometriosis-related fibrosis, leading to an unrecognizable appearance, confusing the cyst with other ovarian neoplasms. This modification could be attributed to the intensive presence of fibroblasts in the cystic and ovarian stromata and the increased TGF-β concentration within the peritoneal fluid, as it’s secreted by both endometriosis and the over-activated macrophages. In fact, endometriosis-induced fibrosis is growing in importance, as it may impair fertility in many ways rather than simply disturbing the normal pelvic anatomy by adhesions. One study has demonstrated the ability of stromal fibrosis in provoking subfertility through restricting the follicular development. Moreover, inhibiting the LH serum levels resulted in the regression of these fibrotic changes and improved folliculogenesis [10]. It is noteworthy that the luteinizing hormone (LH) can also induce the expression of TGF-β in the ovarian interstitial cells [11] and subsequently provokes the stromal fibrosis. Similarly, a Cochrane review has also demonstrated that inhibiting the LH levels has a significant positive influence on the results of in-vitro fertilization in patients with endometriosis [12], which may be explained by the reduction of the fibrotic changes within the ovarian stroma by inhibiting the stromal expression of TGF-β. These findings collectively suggest that increased TGF-β concentrations in endometriotic patients may play a prospective role in ovarian stromal fibrosis and infertility. We believe that the mural fibrosis that was noticed in our case reflects some of the TGF-β influence on the ovarian stroma.

However, this rare morphology has hardened the intra-operative diagnosis of the cyst, and confused it with many other ovarian neoplasms. Initially, ovarian stromal tumors – such as fibromas and thecomas – were suspected since they have a higher occurrence in the third decade of life [13]. In addition, these tumors also manifest as rough white masses during laparoscopic exploration, and they can be precisely diagnosed by pathologic examination. However, the excised cyst in our case contained a cavitation that made it inconsistent with ovarian stromal tumors that are usually solid.

In cases where patients are seeking fertility, surgical management should aim at minimizing the ovarian cortical damage by applying minimally invasive procedures. Therefore, avoiding the electrocoagulation of the ovarian cortex and performing a cystectomy with minimal damage to the ovarian tissue are considered to be the optimal management [14].

## 4 Conclusion

Tissue fibrosis is a potential complication of endometriosis and it is induced mainly by the elevated level of TGF-β within the peritoneal fluid. We believe that the mural fibrosis seen in the serous cyst reflects the endometriosis’ ability to provoke fibrosis within the ovarian stroma, because both cystic and ovarian stromata consist of the same cellular component (fibroblasts), and are present in the same environment, which suggests that both stromata will respond similarly to the elevated level of TGF-β, and ultimately, ovarian stromal fibrosis happens. In conclusion, this mechanism may play a potential role in provoking infertility in patients with endometriosis. However, it could be reversed by administering the LH inhibiting regimens that was described in the previously mentioned Cochrane review.

## Informed consent

Written patient consent was taken before reporting this case and sharing the surgical and pathologic images.

## Provenance and peer review

Not commissioned, externally peer-reviewed.

## Funding

There were no sources of funding.

## Ethical approval

No ethical approval was needed as we reported the morphology of a serous ovarian cyst that was found incidentally during a classic laparoscopy, neither experimental treatments nor new surgical techniques were used.

## Consent

A written consent was obtained from the patient for publication of this case report and accompanying images.

## Author contribution

Antoine Naem: Reviewed the literature, wrote the entire manuscript, designed the figures, and wrote the figure’s legends.

Lamia Kouba: Reviewed the literature, revised the written language of the manuscript and rephrased many sentences.

Bashar Al-Kurdy: Lead the surgical procedure, supervised the writing of the manuscript, and revised the manuscript

## Registration of research studies

N/A.

## Guarantor

Mr. Antoine Naem.

## Declaration of Competing Interest

All of the authors declared that they have no conflict of interest.
